# Self-compassion moderates the perfectionism and depression link in both adolescence and adulthood

**DOI:** 10.1371/journal.pone.0192022

**Published:** 2018-02-21

**Authors:** Madeleine Ferrari, Keong Yap, Nicole Scott, Danielle A. Einstein, Joseph Ciarrochi

**Affiliations:** 1 School of Psychology, Australian Catholic University, Sydney, New South Wales, Australia; 2 School of Psychology, University of Sydney, Sydney, New South Wales, Australia; 3 School of Health Sciences, RMIT University, Bundoora, Victoria, Australia; 4 Department of Psychology, Macquarie University, Sydney, New South Wales, Australia; 5 Institute of Positive Psychology and Education, Australian Catholic University, Sydney, New South Wales, Australia; Maastricht University, NETHERLANDS

## Abstract

**Background:**

Psychological practitioners often seek to directly change the form or frequency of clients’ maladaptive perfectionist thoughts, because such thoughts predict future depression. Indirect strategies, such as self-compassion interventions, that seek to change clients’ relationships to difficult thoughts, rather than trying to change the thoughts directly could be just as effective. This study aimed to investigate whether self-compassion moderated, or weakened, the relationship between high perfectionism and high depression symptoms in both adolescence and adulthood.

**Methods:**

The present study utilised anonymous self-report questionnaires to assess maladaptive perfectionism, depression, and self-compassion across two samples covering much of the lifespan. Questionnaires were administered in a high school setting for the adolescent sample (Study 1, *M*_*age*_ = 14.1 years, *n* = 541), and advertised through university and widely online to attract a convenience sample of adults (Study 2, *M*_*age*_ = 25.22 years, *n* = 515).

**Results:**

Moderation analyses revealed that self-compassion reduced the strength of relationship between maladaptive perfectionism and depression in our adolescent Study 1 (*β* = -.15, *p* < .001, R^2^ = .021.) and our adult study 2 (*β* = -.14, *p* < .001, R^2^ = .020).

**Limitations:**

Cross-sectional self-reported data restricts the application of causal conclusions and also relies on accurate self-awareness and willingness to respond to questionnaire openly.

**Conclusions:**

The replication of this finding in two samples and across different age-appropriate measures suggests that self-compassion does moderate the link between perfectionism and depression. Self-compassion interventions may be a useful way to undermine the effects of maladaptive perfectionism, but future experimental or intervention research is needed to fully assess this important possibility.

## Introduction

The increasing prevalence of depression is widely considered a global health epidemic [1]. Estimates suggest that depression costs 1% of the total gross domestic product (GDP) in Western nations [2]. Depressive symptoms cause a profound and insidious burden to both the individual (i.e., daily functioning and quality of life) and broader society (i.e., strain on healthcare systems, and loss of production through work absenteeism, early retirement and premature mortality) [3]. This extensive burden emphasises the urgency of evidence-based detection, prevention and treatment initiatives for depression. A primary mechanism underpinning the development and maintenance of depression is perfectionism. Perfectionistic trepidations, particularly those shaped by social influences such as perceived demands of perfection from others and concern over mistakes, may potentially exacerbate daily stresses and create a vulnerability toward depression [4].

Whilst this perfectionism-depression link has been widely documented [5, 6], potential moderators of this link remain under-researched. The present research focused on the possibility of self-compassion [7], a healthy way of relating to oneself, as a moderator. Given past research suggests that some cognitive strategies helpful to adults may be less useful for youth [8], we adopted a lifespan approach and assessed moderation in separate adolescent and adult samples.

### The perfectionism-depression link

Defined as setting extremely high standards, and accompanied by a highly critical evaluation of the self in pursuit of these standards, perfectionism is a complex multidimensional construct [9]. Several studies have shown that the striving to attain high personal standards in and of itself is not necessarily destructive, and can have adaptive and positive consequences [10]. This healthy form of perfectionism, also called ‘personal strivings perfectionism’ is not related to psychopathology, and instead predicts higher levels of conscientiousness, self-esteem, and positive affect as well as lower depression and perceived hassles [10]. By contrast, perfectionism that involves self-criticism, concerns about making mistakes, and concerns about being negatively evaluated by others has been linked to various forms of psychopathology [11, 12]. This component of perfectionism has been labelled maladaptive perfectionism [13], and is the focus of the current study.

Accumulating research has linked perfectionism to depressive symptoms across both clinical [14] and community samples [15]. Longitudinal studies have shown the temporal precedence of self-critical perfectionism before depressive symptomology [16]. In addition, perfectionism has been shown to predict the development of later episodes of major depressive episodes [17]. A recent meta-analysis of longitudinal studies found that perfectionism at baseline predicted later change in depression symptoms beyond neuroticism, thus lending credence to the conceptualisation of perfectionism as a premorbid personality type which increases vulnerability to depression [4]. Indeed, perfectionism has been identified as a trans-diagnostic construct which underlies and maintains many forms of psychopathology including eating disorders, anxiety and even schizophrenia [11].

### Perfectionism across the lifespan

In recent years, clinicians and researchers are interested in how perfectionism develops and manifests itself across the developmental lifespan [18, 19]. A robust correlation between depression and socially prescribed perfectionism (SPP) has been found in adults [6]. SPP reflects a type of maladaptive perfectionism grounded in an external locus of control, strong needs for approval and fear of negative evaluation, and in student and clinical samples is closely linked to depression as measured by the Beck Depression Inventory [17, 20]. Typically the excessively high and unattainable goals of adults with SPP tend to result in perceived failure, fostering negative self-evaluation which may evolve into depression [18].

Evidence has accumulated to support the hypothesis that socially prescribed perfectionism is also damaging in adolescence. Einstein, Lovibond and Gaston [21] observed significant correlations between SPP and both depression (*r* = .37) and anxiety (*r* = .32) before a set of final year high school exams. In introductory psychology university students, Flett, Blankstein and Hewitt [22] observed negative relationships between SPP and test performance (*r* = -.27); and between SPP and negative affect after sitting a test (*r* = .30). In comparison, self-oriented perfectionism was neither correlated with depression nor anxiety in either study [21, 22]. Whilst the bulk of this research in adolescent samples is cross-sectional in design, a small number of longitudinal studies lend greater support to the causal relationship of perfectionism on depression symptoms. One adolescent study found that that perfectionistic tendencies predict depressive symptoms one year later [23]. Another RCT for clinically depressed adolescents found that compared to low perfectionism, high baseline perfectionism predicted elevated depression scores 12 weeks in both control and intervention groups [24]. Interestingly, a three-year longitudinal study in high school students found the reverse; that depressive symptoms predicted an increase in maladaptive perfectionism over time [25]. Whilst the exact direction of the perfectionism-depression link requires further clarification, research suggests that adolescents are prone to internalising expectations of others [26–28], potentially more so than adult populations. Thus it is important to consider the context of the developmental lifespan when understanding the link between perfectionism and depression.

### Self-compassion; a healthy way of relating to oneself

Self-compassion, a healthy way of relating to oneself which can be cultivated [7], is a construct attracting increasing attention in clinical psychology and has only tentatively been examined in the context of perfectionism and depression. Self-compassion is defined as ‘being open to and moved by one’s own suffering, experiencing feelings of caring and kindness toward oneself, taking an understanding, nonjudgmental attitude toward one’s inadequacies and failures, and recognizing that one’s own experience is part of the common human experience’ (p.244, [29]). Neff proposed that there are three basic components of self-compassion: self-kindness (as opposed to self-criticism), common humanity (as opposed to separating and isolating oneself), and mindfulness (as opposed to over-identifying with one's painful experiences) [29]. Neff also suggested that self-compassion requires individuals to take on a mindful perspective in which thoughts and feelings are experienced without judgment. Instead of avoiding or trying to counteract painful experiences, self-compassion enables individuals to be open to difficult emotions, extend kindness toward themselves, and recognize that these experiences are common to humanity so that they are not alone in their pain or suffering. Individuals who are self-compassionate recognize that imperfections, faults, and difficulties in life are universal and are therefore less likely to be self-critical, harsh, and judgmental [7].

Theoretically, as self-compassion fosters self-kindness and acceptance of self, such healthy ways of relating to oneself should directly buffer the negative effects of perfectionism. This may particularly be the case should people respond with self-compassion in times of error or distress rather than harsh self-criticism which could otherwise lead to maladaptive helplessness and hopelessness. One study to-date has specifically examined self-compassion in the context of perfectionism and depression [30]. The authors found that self-compassion partially mediated the link between perfectionism and depression in a sample of university students [30]. Positioning self-compassion to be a mediator theoretically suggests that the perfectionism reduces self-compassion and reduced self-compassion in turn increases depression. In contrast, we focused on moderation and the possibility that the link between perfectionism and depression would be influenced by self-compassion.

### Moderation and the perfectionism-depression link

**Fig 1:**
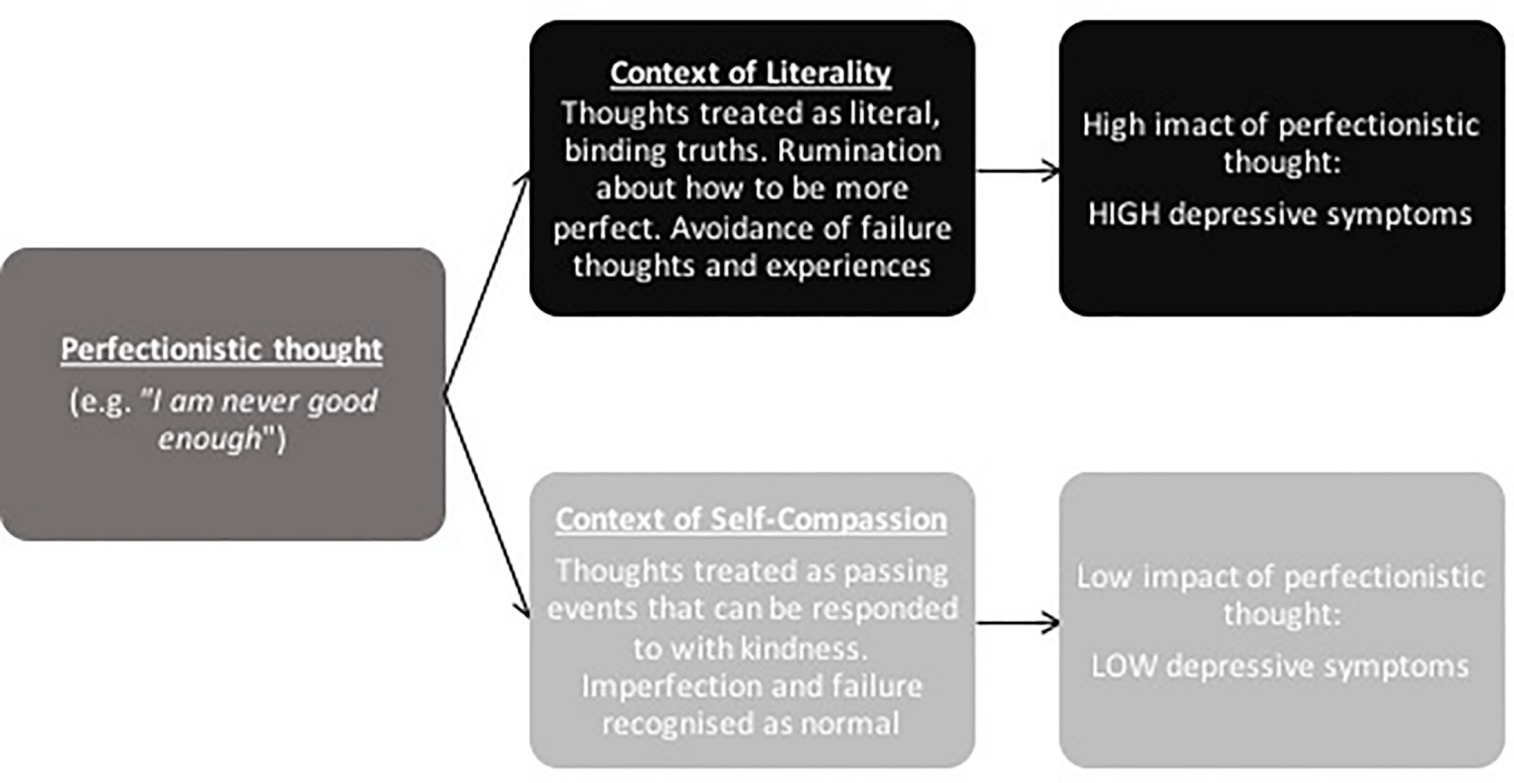
Theoretical model of the proposed moderating role of self-compassion on the perfectionism-depression link.

Theory suggests two ways to combat maladaptive perfectionism [31], as illustrated in Fig 1. First, practitioners may seek to alter the form or frequency of the perfectionistic thought, as when they discourage the parents of youth from insisting young people strive for excessively high standards. In Fig 1, this would be equivalent to preventing the “I must always be perfect” thought from occurring in the first place. Or the practitioner may seek to challenge the thought using cognitive reappraisal. However, recent research suggests that such cognitive reappraisal strategies sometimes fail, especially with young people [8, 32].

A second way to combat maladaptive perfectionism is to alter the person’s relationship to a thought. As illustrated in the top path of Fig 1, one may increase the impact of a perfectionist thought by treating it as literally true. For example, if a child says “I am never good enough”, a parent could create a “context of literality” by saying, “that is because you never try hard enough”. The parent is teaching the child that the thought is true and should be responded to. In contrast, the parent may also reduce reactivity to the thought (lower path, Fig 1) by saying, “I know how you feel. I sometimes feel I am not good enough too, but I know that is just a thought in my head. Everybody makes mistakes”. In this later context, the young person is taught to experience the perfectionistic thought as a normal, passing event that can be responded to with self-compassion, rather than as a binding “truth” that dictates how one “must” feel and acts [33]. This theorising suggests a moderation hypothesis, namely, that self-compassion will influence the strength of the link between maladaptive perfectionism and depression.

Several studies have sought to identify potential moderators of the perfectionism-depression link. For example, perceived social support and social connectedness has been shown to moderate the relationship between maladaptive perfectionism and depression [34, 35]. While associated, these constructs do not overlap conceptually with self-compassion as they depend on the perception and availability of external social resources. Flett, Besser, Hewitt and Davis [36] found a sub-component of self-silencing called externalized self-perception that acted as a moderator, such that the positive relationship between socially-prescribed perfectionism and depression was much stronger for individuals with high levels of externalized self-perception. Like individuals with low self-compassion, individuals with high externalized self-perception are not accepting of their own flaws and depend on the approval of others before they can accept themselves.

### Aims and hypotheses

Theory suggests that self-compassion should lessen the negative impact of self-critical thoughts such as those associated with maladaptive perfectionism (see Fig 1). Thus, we sought to test the moderating effect of self-compassion on the perfectionism-depression link. In addition, we sought to adopt a lifespan perspective, investigating the potential moderating role of self-compassion for both adolescents and adult populations. We hypothesized that self-compassion would weaken, or “decouple”, the link between maladaptive perfectionism and depression. Specifically, we predicted that the relationship between perfectionism and depression would be stronger for adolescents and adults with lower self-compassion compared to those with higher self-compassion. Given past research suggests that some cognitive strategies, such as reappraisal, may be less effective for youth, we explored the extent that any moderation effects were consistent in adolescent and adult samples. Moderation analysis allows for the examination of how the strength of the relationship between perfectionism and depressive symptoms may change at different levels of self-compassion (low, medium and high).

## Study 1: Self-compassion in adolescence

### Method

#### Participants

Five hundred and forty-one adolescents (99 male, 442 female) from grades 7 to 10 were recruited from five Australian private schools including three female, one male and one co-educational school. Participants completed the questionnaires as part of the baseline assessment for a larger wellbeing intervention study. Given the data presented here is baseline, it was not affected by the intervention and reflects general functioning. Their mean age was 14 years and 1 month (*range =* 12 years 7 months to 15 years 6 months) and 14 participants did not report their age. The majority of students were born in Australia (*N* = 484, 59.5%), the next most common country of birth being China (*N* = 15, 2.8%) and England (*N* = 6, 1.1%). The majority also spoke English as their first language (*N* = 508, 93.9%).

#### Materials

The Child and Adolescent Perfectionism Scale (CAPS)[37] is a 22 item questionnaire that assesses both self-oriented perfectionism (SOP, i.e. high self-standards) and socially prescribed perfectionism (SPP, i.e. perceived demands of perfection from others). The CAPS is the most widely used multidimensional measure for child and adolescent populations, both inpatient and community [38], and has demonstrated good factor structure and temporal stability [39]. The SPP subscale was used in the current study as a measure of maladaptive perfectionism. Adolescents rated each item on a 5-point Likert scale, (1 = *False–not at all true of me*, 5 = *Very true of me*). The internal consistency for the maladaptive perfectionism measure in the current study was high (*α* = .92).

The Short Mood and Feelings Questionnaire (SMFQ) [40] is a 13 item scale which assesses low mood and related psychological constructs such as low self-esteem and self-worth, herein referred to as depressive symptoms. The SMFQ has successfully been used as a measure of depressive symptoms in community samples including adolescents aged 10–13 years [41] and 10–15 years [42–44]. Rhew, Simpson, Tracy, Lymp, McCauley, Tsuang, et al. [45] found that the child-reported version of the SMFQ had reasonable diagnostic accuracy for the presence of a depressive disorder in adolescents aged 10–13 years according to receiver operating characteristic curve analyses (AUC = 0.73). The 13 items utilise a 3-point Likert scale, (0 = *Not True*, 2 = *Very true*). The adolescent must rate whether the provided phrase (*i*.*e*. *‘I felt so tired I just sat around and did nothing’)*, is indicative of their feelings and actions over the timeframe of the previous two weeks. The internal consistency of this scale was high in the present study (*α* = .91).

The Self-Compassion Scale–Short form (SCS-s) [46] is a 12 item questionnaire developed to assess one’s “ability to hold feelings of suffering with a sense of warmth, connection and concern” (p.226, [47]). The subscales include self-kindness (*i*.*e*. *‘I try to be loving towards myself when I’m feeling emotional pain’)*, common humanity (*i*.*e*. *‘I try to see my failings as part of the human condition’)* and mindfulness *(i*.*e*. *‘When something upsets me I try to keep my emotions in balance’)*. Adolescents respond to questions using a 5-point Likert style (1 = *Almost never*, 5 = *Almost always*). The SCS-s has been used in large Australian adolescent samples (*α* = 0.75) [31], and the current internal consistency was high (*α* = .80).

#### Procedure

Approval was granted by two university Human Research Ethics Committees; Macquarie University (reference code: 5201500115) and the Australian Catholic University (reference code: 0000019858) for Study 1. Participants completed the online questionnaire using the online platform Qualtrics. The adolescents completed the questionnaires during school hours as part of the baseline assessment for a larger wellbeing intervention study [48]. Students were supervised by their class teachers whilst completing the questionnaire online using electronic tablets. This sample was a subset of the data collected for the larger wellbeing intervention study [48], available at the time of analysis as the larger project is ongoing. Participation was voluntary and anonymous, and both students and their parents completed consent forms. All students enrolled in participating grades and schools were invited to join the study, schools opted for a passive or active consent procedure. In the passive procedure, parents were notified of the intention to conduct surveys and all students in the year completed surveys. In the active procedure, only students who returned completed information and consent forms completed surveys. Irrespective of whether a passive or active consent procedure was followed for the administration of surveys, participants were only included in the current research data analyses for the present study if both students and parents also completed an information and consent form. There were no further inclusion criteria.

At the conclusion of the online questionnaires, all participants were provided with free and readily accessible telephone counselling phone numbers and website links for support. Students were also encouraged to link in with their school counsellor or teacher in the event that questions in the survey had caused discomfort or distress. In addition, all data collected was re-identifiable through a code maintained at the school by a designated senior staff member. Students whose responses indicated they may be at risk of harm to self or others were identified by the research team. The chief investigator then liaised with the designated staff coordinator in order to break the code to identify the student, and the school followed their wellbeing protocol in order to arrange a follow-up check in with the participant and assess the potential need for future referral. This process for breaking confidentiality in this way was explained in the information and consent form.

#### Analyses

In both studies, we assessed moderation by conducting hierarchical multiple regression as recommended by Aiken and West [49] using the Preacher and Hayes PROCESS macros [50]. To avoid multicollinearity issues, we centered the predictor variables prior to the analyses. The dependent variable was depression and predictor variables included maladaptive perfectionism (MP), and self-compassion (entered in the first step). The product term (MP * self-compassion) was entered in the second step to examine the change in variance resulting from the interaction between MP and self-compassion.

## Results

### Preliminary analysis

In total, 1,249 potential participants who were enrolled in the involved high-schools were invited to participate. Of these, 574 provided informed consent from both the adolescent and their primary caregiver (approximately 46%). All adolescent participants opened the link to the questionnaire, and 14 dropped out before completing the first scale and participants who did not complete at least one scale were removed (*N =* 19). Of the remaining participants, Little’s MCAR test showed that any missing data was at random (Little’s MCAR test χ^2^ = 3.26, *df* = 3, *p* = 0.35). The final sample size for the current study was 541.

Normality testing revealed that maladaptive perfectionism and self-compassion scores were normally distributed, while depression scores were positively skewed which is not surprising given the non-clinical nature of the community sample. However, as the sample size was large, transformation of the data did not produce any differences in results and therefore, non-transformed data was used in all analyses [51]. Assumptions for all multiple regression analyses were met. Descriptive statistics for all variables are presented in Table 1. There was a significant difference between genders in depression scores (males *M* = 5.05, *SD* = 4.94, females *M* = 7.34, *SD* = 6.13, *t*(539) = -3.47, *p* < .001). There was no significant difference for self-compassion (males *M* = 3.20, *SD* = .54, females *M* = 3.12, *SD* = .62, *t*(539) = 1.26, *p* = .21) and maladaptive perfectionism (males *M* = 24.47, *SD* = 8.22, females *M* = 25.57, *SD* = 9.35, *t*(539) = -1.08, *p* = .28). A significant but very weak correlation between age and depression (*r*^2^ = .09, *p* < .05) and self-compassion (*r*^2^ = .11, *p* < .05) was found.

**Table 1:** Intercorrelations, means, and standard deviations for the adolescent sample (*n* = 541).

Variable	*M*	*SD*	1	2	3
1. Maladaptive perfectionism (CAPS)	25.37	9.16	-		
2. Depression (SMFQ)	6.92	5.99	.45**	-	
3. Self-compassion (SCS-s)	3.13	.61	-.49**	-.63**	-

** *p* < .001

### Main analyses

Correlations between depression, maladaptive perfectionism, and self-compassion scales are presented in Table 1. Results showed a strong a positive relationship between maladaptive perfectionism and depression, and a strong negative relationship between self-compassion and depression. Risk of collinearity was deemed to be unlikely given the Variance Inflation Factors (VIF) was within acceptable limits, i.e., less than 5 [49](see Table 2).

**Table 2:** Hierarchical regression analysis predicting depression (SMFQ) and probing the interaction between self-compassion (SCS-s) and perfectionism (CAPS; n = 541) for the adolescent sample.

	*B*	SE *B*	*β*	*sr*^*2*^^¶^	*p-*value	95% CI	VIF
Step 1							
Self-Compassion	-5.29	.37	-.53	-.52	< .001	-6.02, -4.56	1.31
Maladaptive Perfectionism	.13	.02	.19	.22	< .001	0.78, 0.18	1.31
Step 2							
Self-Compassion	-5.51	.37	-.56	-.54	< .001	-6.23, -4.79	1.33
Maladaptive Perfectionism	.11	.02	.17	.20	< .001	0.07, 0.16	1.33
Self-Compassion * Maladaptive Perfectionism	-.14	.03	-.15	-.19	< .001	-0.20, -0.08	1.02

^¶^*sr*^*2*^ denotes squared semi-partial coefficient.

Model 1: *R*^*2*^ = 0.43, *adjusted R*^*2*^ = 0.42, *F*(2,538) = 197.51, *p* < .001.

Model 2: *R*^*2*^ = 0.45, *adjusted R*^*2*^ = 0.44, *F*(3,537) = 143.26, *p* < .001.

### Moderation analysis

To assess moderation, we conducted hierarchical multiple regression using the Preacher and Hayes PROCESS macros [50]. Maladaptive perfectionism, self-compassion and their interaction was used to predict depression. To avoid multicollinearity issues, the predictor variables are centred prior to regression analyses.

**Fig 2:**
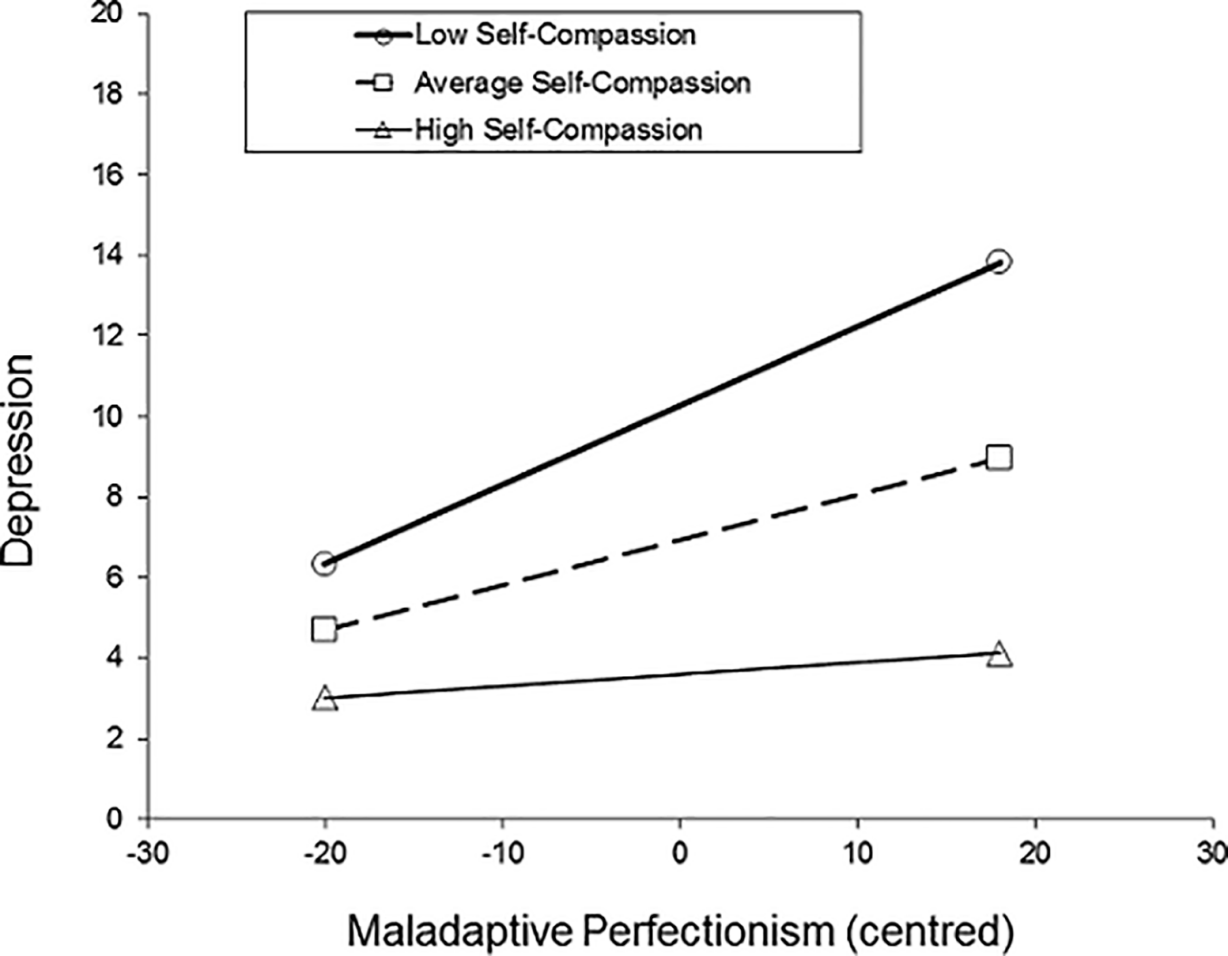
Adolescent sample. The moderating effect of self-compassion (SCS-s) on perfectionism (CAPS) and depression (SMFQ) scores (n = 541).

The analysis showed that inclusion of the product term into the model resulted in a significant increase in variance accounted for by the predictor variables, Δ*R*^*2*^ = .021, Δ*F* (1, 537) = 20.456, *p* < .001. This indicated that self-compassion significantly moderated the relationship between maladaptive perfectionism and depression (see Table 2). To probe the significant interaction, a series of simple regression equations were generated. Following Aiken and West’s [49] suggestion, three simple regression equations were calculated at different levels of self-compassion: (1) one standard deviation below the mean total self-compassion score (2) the mean total self-compassion score, and (3) one standard deviation above the total self-compassion score (see Fig 2). The plot of the interaction showed a buffering effect of self-compassion; there was a weaker, non-significant positive relationship between maladaptive perfectionism and depression for individuals with high self-compassion compared to those who had low self-compassion. Simple slopes analyses showed that the relationship between perfectionism and depression was significant at one standard deviation below the mean self-compassion score (b = 0.20, *t* = 6.87, *p* < .0001, 95% CI: 0.14, 0.25), and at the mean self-compassion score (b = 0.11, *t* = 4.63, *p* < .0001, 95% CI: 0.07, 0.16). However, the slope at one standard deviation above the mean self-compassion score was not significant (b = 0.03, *t* = 0.87, *p* = 0.38, 95% CI: -0.04, 0.09).

When the moderation analyses were re-run controlling for age and gender as covariates, there were no notable differences in the results (see online Supporting Information files). Consequently, the results for the analyses without the included covariates are reported here.

## Study 2: Self-compassion in adults

### Method

#### Participants

The sample for Study two consisted of 515 adults from the general population who were recruited through word of mouth and snowball sampling. The inclusion criteria for this study was that participants were aged over 18 years and had a level of English that allowed for completion of the online survey independently. Given the online advertising strategy, we were unable to ascertain the number of potential participants approached. The final sample consisted of 158 males and 357 females. Participants were aged between 18 and 72 years, (*M* = 25.22, *SD* = 9.19). Participation was voluntary and anonymous. No incentive to participate in the current study was offered to participants, beyond making a contribution to research. In relation to ethical considerations, contact could not be made to participants who scored high on depression as the survey was anonymous. However, the investigators' contact details and information about free telephone counselling were provided in the study's plain language information statement for participants who felt distress or discomfort when completing the survey.

#### Materials

Perfectionism was measured using the combined score of four sub-scales from the Multidimensional Perfectionism Scale [9], as recommended by Kawamura and Frost [52]. These sub-scales were: Concern over Mistakes, Parental Expectations, Parental Criticism and Doubts about Actions. Adults rated each item on a 5-point Likert scale, (1 = *strongly disagree*, 5 = *strongly agree*). Previous studies have found this measure to have high internal consistency (*α* = .91) [52], and strong validity with other perfectionism measures [53, 54]. The internal consistency for the maladaptive perfectionism measure in the current study was high (*α* = .93).

The Depression sub-scale from the 21-item Depression, Anxiety and Stress Scale (DASS-21)[55] was used to measure depressive symptoms. The Depression scale of the DASS-21 contains seven items. Adults used a 4-point Likert scale to indicate how frequently each item had occurred during the past week, (0 = *did not apply to me at all*, 3 = *applied to me very much*, *or most of the time*). This scale has demonstrated strong convergent and divergent validity for the depression subscale in both clinical [56] and community samples [57]. Internal consistency of the Depression subscale in the current study was high (*α* = .93).

The Self-Compassion Scale [29] was used to measure levels of self-compassion. This scale consists of 26 items measuring the components of self-compassion; Self-Kindness, Common Humanity, and Mindfulness. Adults rated each item on a 5-point Likert scale (1 = *almost never*, 5 = *almost always*). The total self-compassion scale score was calculated using the mean of scores on all items after the scores on negatively worded items were reversed. Previous studies have shown that the self-compassion scale (SCS) has very good internal consistency, *α* = .94 [29]. In the present study, the total SCS had excellent internal consistency (*α* = .94).

#### Procedure

Approval was granted for Study 2 by the RMIT Human Research Ethics Committee (application number: BSEHAPP 08–11). Information about the study and links to the online survey were publicised on Facebook, a social networking site. The advertisement was shared through the friend network of the researchers and friends were invited to share the advertisement on their friendship networks, creating a snowball recruitment effect. The online questionnaire was administered using the Survey Monkey platform, and participants were provided with an electronic information and consent form prior to commencing the questionnaires. The order of the questionnaires was randomized for each participant to minimize order effects.

## Results

### Preliminary analysis

The study was advertised online to an undetermined number of potential participants. Six hundred and fifteen participants opened the survey link, and participants who did not complete at least one scale were removed prior to analysis (*N* = 100), resulting in a final sample of 515. Normality testing revealed that maladaptive perfectionism and self-compassion were normally distributed, whereas not surprisingly given the community sample, depression was positively skewed. However, as the sample size was large, transformation of the data did not produce any differences in results and therefore, for each of interpretation, non-transformed data was used in all analyses. Assumptions for all multiple regression analyses were met. Descriptive statistics for all variables are presented in Table 3. There was a significant difference between genders in depression scores (males *M* = 12.85, *SD* = 5.18, females *M* = 13.97, *SD* = 6.30, *t(*513) = -1.96, *p* = .05), and self-compassion scores (males *M* = 3.05, *SD* = .72, females *M* = 2.72, *SD* = .77, *t(*513) = 4.52, *p* < .001). A significant positive correlation was also found between age and maladaptive perfectionism (*r* = .12, *p* = .005). The inclusion of age as a covariate did not have any significant effect on results and as such, it was excluded from the analyses.

**Table 3:** Intercorrelations, means, and standard deviations for the adult sample (n = 515).

Variable	*M*	*SD*	1	2	3
1. Maladaptive perfectionism (MPS)	60.53	16.83	-		
2. Depression (DASS subscale)	13.63	6.00	.58**	-	
3. Self-compassion (SCS)	2.82	.77	-.63**	-.62**	-

** *p* < .001

### Main analyses

Correlations between depression, maladaptive perfectionism, and self-compassion scales are presented in Table 3. Results showed a strong a positive relationship between maladaptive perfectionism and depression, and a strong negative relationship between self-compassion and depression. Risk of collinearity was deemed to be unlikely given the satisfactory Variance Inflation Factors (VIF) statistic (see Table 4).

**Table 4:** Hierarchical regression analysis predicting depression (DASS) and probing the interaction between self-compassion (SCS) and maladaptive perfectionism (MPS) in adults (n = 515).

	*B*	SE *B*	*β*	*sr*^*2*^^¶^	*p-*value	95% CI	VIF
Model 1							
Self-Compassion	-6.66	.666	-.425	-.329	< .001	-7.97, -5.35	1.67
Maladaptive Perfectionism	.221	.030	.310	.240	< .001	0.16, 2.8	1.67
Model 2							
Self-Compassion	-7.09	.662	-.453	-.347	< .001	-8.39, -5.79	1.71
Maladaptive Perfectionism	.206	.030	.289	.222	< .001	0.15, 0.27	1.69
Self-Compassion * Maladaptive Perfectionism	-.128	.029	-.144	-.142	< .001	-0.19, -0.07	1.02

^¶^*sr*^*2*^ denotes squared semi-partial coefficient.

Model 1: *R*^*2*^ = 0.45, *adjusted R*^*2*^ = 0.44, *F*(2, 512) = 204.99, *p* < .001.

Model 2: *R*^*2*^ = 0.45, *adjusted R*^*2*^ = 0.44, *F*(2, 512) = 204.99, *p* < .001

### Moderation analysis

**Fig 3:**
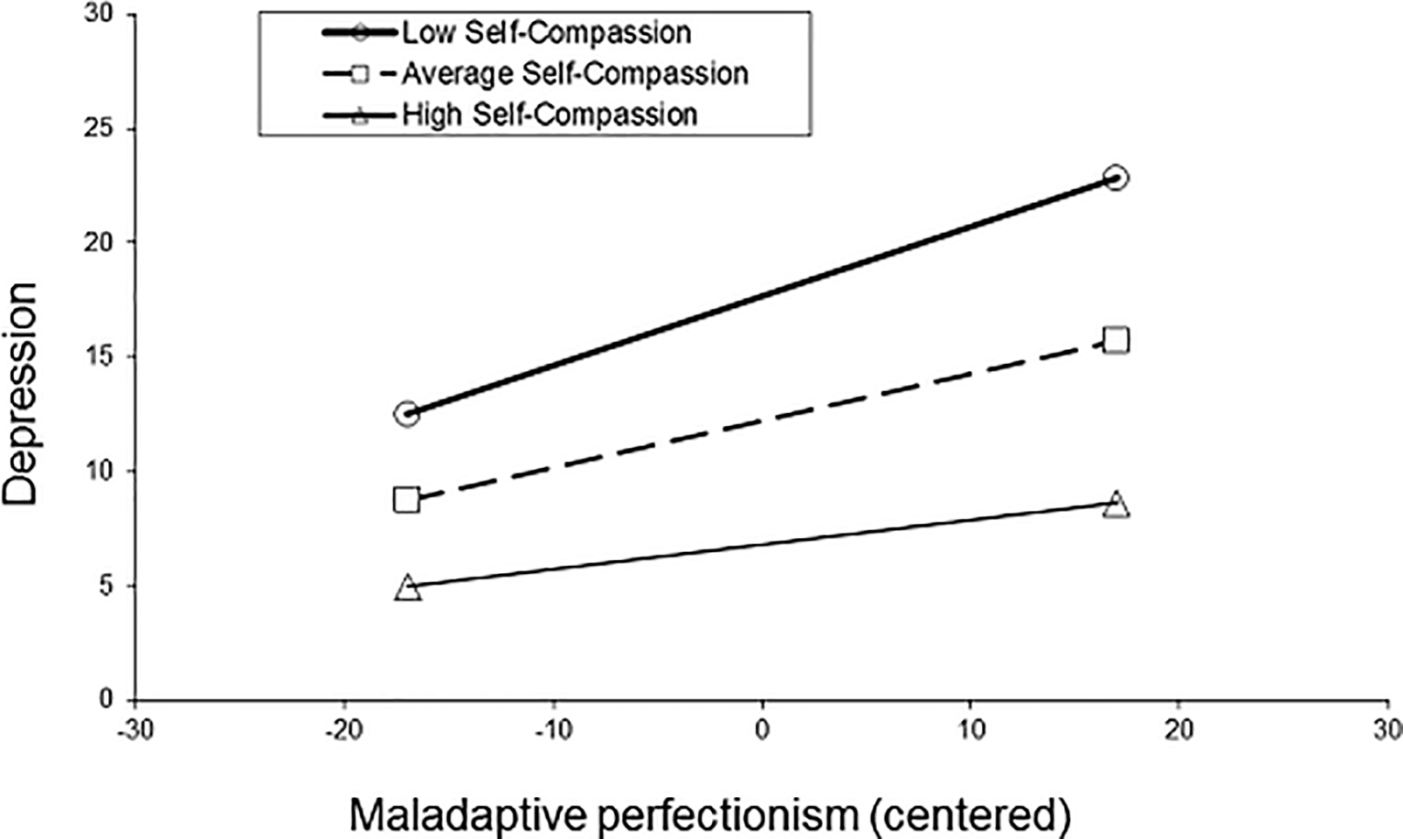
Adult sample. The moderating effect of self-compassion (SCS) on perfectionism (MPS) and depression (DASS- Depression subscale) scores (n = 515).

Table 4 presents results of the hierarchical multiple regression. The analysis showed that inclusion of the product term into the model resulted in a significant increase in variance accounted for by the predictor variables, Δ*R*^*2*^ = .020, Δ*F* (1, 511) = 19.26, *p* < .001. This indicated that self-compassion significantly moderated the relationship between maladaptive perfectionism and depression. To probe the significant interaction, a series of simple regression equations were generated. Following Aiken and West’s [49] suggestion, three simple regression equations were calculated at different levels of self-compassion: (1) one standard deviation below the mean total self-compassion score (2) the mean total self-compassion score, and (3) one standard deviation above the total self-compassion score (see Fig 3).

The plot of the interaction showed a buffering effect of self-compassion; whilst still significant there was a weaker positive relationship between maladaptive perfectionism and depression for individuals with high self-compassion compared to those who had low self-compassion. Simple slopes analyses showed that the relationship between perfectionism and depression was significant at one standard deviation below the mean self-compassion score (b = 0.30, *t* = 8.62, *p* < .0001, 95% CI: 0.24, 0.37), and at the mean self-compassion score (b = 0.21, *t* = 6.87, *p* < .0001, 95% CI: 0.15, 0.27). The slope at one standard deviation above the mean self-compassion score, whilst smaller, was also significant at *p =* 0.05 (b = 0.11, *t* = 2.73, *p* = 0.007, 95% CI: 0.03, 0.19).

Similar to study 1, when the moderation analyses were re-run controlling for age and gender as covariates, there were no notable differences in the results (see results in online supplementary materials). Consequently, the results for the analyses without the included covariates are reported here.

## Discussion

The findings of both the adolescent and adult samples support our initial hypotheses. Consistent with our main hypothesis, self-compassion moderated the maladaptive perfectionism-depression link such that self-compassion weakened the relationship between maladaptive perfectionism and negative affect. Second, although Studies 1 and 2 differed in terms of sample and measures used, we found the size of the moderation effect involving to be virtually identical in the adolescent (*β* = -.15) and adult (*β* = -.14) samples. Whilst significant and consistent findings were obtained in the current study, it is important to acknowledge these effect sizes would be considered in the small range (2% of variance) explained by the moderation effect). Thus whilst self-compassion does appear to be a significant moderator, it does not seem to completely explain existing variance in the perfectionism-depression link.

Despite this, our findings are in keeping with previous studies that have found significant correlations between maladaptive perfectionism, depression, and self-compassion [7, 58]. The current findings deepen our understanding of the previously found link between maladaptive perfectionism and depression [47, 59, 60]. The current study is unique in that it is the first to show that self-compassion can buffer the effects of maladaptive perfectionism on depression. Both adults and adolescents who had high levels of self-compassion were not as influenced by maladaptive perfectionism compared to their counterparts with low self-compassion.

### Theoretical implications of the current study

The moderating role of self-compassion on the link between perfectionism and depression has important theoretical implications. The current results support the ‘compassionate context’ model, as proposed by Marshall, Parker, Ciarrochi, Sahdra, Jackson and Heaven [31] (see Fig 1). This theoretical model aligns with an acceptance and commitment therapy (ACT) approach, positing that it is not the form of the thought that is critical; it is the function. That is, it is not the presence of perfectionistic thoughts that is expected to cause depression (form), but rather the way people *react* to these thoughts that matters. Our results suggest that reacting to perfectionistic thoughts with self-compassion reduces the effect of these thoughts from developing into negative affect. Comparatively, reacting with a lack of self-compassion is likely to include self-criticism, isolation and fusion with unhelpful thoughts. This alternative context is expected to feed into the maladaptive nature of perfectionism and contribute to the development of depression symptoms.

This theoretical approach may be a helpful way to understand the perfectionism-depression link in a modern context whereby many aspects of our globalised culture frequently elicit maladaptive perfectionistic thoughts. Adolescents and adults alike are frequently exposed to pressures to perform at excessively high standards at school or work, accomplish demanding goals in activities such as sport or music, and have the perfect physical appearance, perfect partner and indeed, the perfect life. Such pressures can encourage people to move between extremes of either maladaptive perfectionism or disengagement and ‘giving up’. Given it is highly likely to be both impractical to constantly challenge and prevent perfectionistic thoughts, self-compassion may offer a healthier way of reacting to and minimising the effects of such thoughts. Self-compassion, though self-kindness, connection and mindfulness, may allow individuals to strive for mastery and goal accomplishment, whilst also holding these goals lightly and demonstrating resilience when adversity arises. This proposed theoretical conceptualisation of how perfectionism leads to depression requires further empirical study, but the present results are at least supportive of this model.

### Clinical implications of the current study

That self-compassion was shown to play a moderating role has practical clinical implications. Self-compassion interventions can be a complement, or perhaps even an alternative to interventions that seek to encourage people to reappraise maladaptive cognitions (e.g., “making a mistake is not horrible, it helps me to learn”). Brockman, Ciarrochi, Parker and Kashdan [8] found that the usefulness of reappraisal varied from person to person and was least likely to be useful for young people. In contrast, we found that self-compassion was equally useful for adolescents and adults.

Individuals with high maladaptive perfectionism are characterised by excessive concerns about being negatively evaluated by others for failing to attain high standards. Likewise, depression is often accompanied by excessive social comparisons and strong beliefs that others are better, more worthwhile and successful. Thus, perfectionistic individuals who suffer from depression are often caught in an unworkable change agenda where attempts to feel better about oneself (through the concealment of perceived failures and the striving for achievements to prove one’s self-worth) invariably fail and further perpetuate the idea of worthlessness. In therapy, they might try to challenge or modify negative evaluative concerns but that paradoxically focuses more attention on their social rank or the lack thereof. It is possible that this pattern of striving in the context of isolation and self-judgment also explains why perfectionism predicts poor therapist alliance and poor response to psychological treatments for depression [61].

As noted by Neff [7], self-compassion is ‘a useful emotion regulation strategy, in which painful or distressing feelings are not avoided but are instead held in awareness with kindness, understanding, and a sense of shared humanity’ (p. 225). Thus, instead of avoiding social comparisons or overcompensating for negative feelings about the self through futile attempts to attain a higher social rank, the cultivation of self-compassion might help individuals to unconditionally accept ones’ failings. Perhaps cultivating self-compassion rather than trying to rid oneself of maladaptive perfectionism might work around the over focus on and fusion with others’ evaluations. Flaws and mistakes are therefore not seen as personal failures but rather evidence of their own humanity and would result in a willingness to reveal to others these flaws and mistakes.

Given the moderating role of self-compassion found in the current study, future clinical intervention research may benefit from assessing the benefits of self-compassion based interventions for perfectionism and depression. Specifically, this could be in the form of self-compassion focused training, or adding components in current psychological therapies for perfectionism. Several preliminary interventions which feature self-compassion have been examined. A recent randomised-control trial (RCT) investigated the efficacy of a Loving-Kindness Mediation (LKM) on self-criticism (*N* = 19) compared to waitlist control (*N* = 19) [62]. Adults who practiced LKM reported significant reductions in self-criticism and depressive symptomology which were sustained at 3-month follow-up [62]. The LKM meditation may be understood to represent a specific component of self-compassion involving mindfulness and self-kindness, and self-criticism is a core component of perfectionism. Thus the results of our study suggest the findings of such previous work [62] may be due to the buffering effect of self-compassion on the link between perfectionism and depression, and point to self-compassion as a potentially parsimonious and effective target intervention.

In addition to self-compassion based meditation, entire intervention programs focusing on self-compassion have also been developed such as Compassion-Focused Therapy [63] for the treatment of psychological disorders and for improving psychological wellbeing [64–67]. In a pilot RCT, Neff and Germer [65] found that participants in a Mindful Self-Compassion program had, by the end of the program, significantly higher self-compassion, mindfulness, and wellbeing, in addition to lower depression, anxiety, stress, and avoidance compared to the waitlist control group. Furthermore, these improvements were maintained at both 6 and 12-month follow-ups. New interventions for adolescents, however, show mixed findings. A 6-week Cognitively-Based Compassion Training (CBCT) course for at-risk youths who had suffered maltreatment reported qualitative benefit and use of strategies in daily life [67]. Across all standardised self-report measures, however, no difference was found compared to waitlist control including anxiety and depression symptoms, emotional regulation or self-harm behaviours [67]. Although there are mixed outcomes for adolescents [67], the results of our studies suggest the potential value of developing and evaluating self-compassion focused interventions for both adolescents and adults. In particular, future self-compassion based interventions should be rigorously tested for their potential efficacy to target perfectionism and the development of depressive symptoms.

### Strengths and limitations

Several limitations were evident in the present study. Firstly, whilst examining two independent age cohorts, the data is cross-sectional and therefore causal conclusions cannot be made. In other words, although the results demonstrated that a negative association existed between depression and self-compassion, it is unclear as to whether self-compassion was the cause or effect of depression. While in our moderation model we suggested that maladaptive perfectionism leads to depression and this effect is buffered by self-compassion, it is also possible depression could precede maladaptive perfectionism [25]. As with most psychological phenomenon, we suspect that the effects are bidirectional. A bi-directional link may also exist between maladaptive perfectionism and low self-compassion. Future longitudinal research is needed to examine this possibility.

Furthermore, whilst significant, the effect sizes for the moderation effects obtained in both studies were relatively small. Future research using structural equation modeling and a prospective longitudinal design would assist in answering this question. Regardless of the direction of effects, our study provides evidence that improving self-compassion might have an impact on maladaptive perfectionism and future research is needed to examine the effects of self-compassion training on maladaptive perfectionism and depression.

Another limitation to our study is the use of online self-report questionnaires. Both studies used age-appropriate scales to investigate the same constructs in both population, however these scales do rely on a degree of self-awareness and willingness to be open and honest. Furthermore, given Study 2 attracted adult participants through online means, this may bias the sample towards individuals who are familiar with and have access to the internet. Study 1 invited year groups at a number of high schools to participate in the questionnaires with a low participation rate (approximately 46%), potentially indicating those who elected to participate may form a biased sample. Furthermore, the schools were all private institutions and located in ACT and NSW, thus results may be specific to these demographics. Furthermore, as participants were from the general population in both studies, generalisations to a clinical population should be made cautiously. Further examination of the moderating effects of self-compassion in a clinical population is required. Finally, both samples included a significantly larger proportion of females (81.7% in study one, 69.3% in study two). Females reported significantly higher depressive symptoms compared to males in both samples, and interestingly males reported higher self-compassion levels only in the adult sample. We did control for gender as a covariate and found little difference in the results compared to our analyses which did not include any covariates. Even so, future research is needed to assess the extent that these findings generalise to males and females.

Whilst acknowledging these limitations, there are several key strengths of the current paper. Assessing the research question in two cohort samples suggests our findings are relevant across the lifespan. Furthermore, as we used different age-appropriate measures to capture the same constructs in the two samples, our consistent findings suggest a strong and robust moderating effect for the underlying construct self-compassion.

### Conclusion

Perfectionism can exact a high psychological cost on individuals and it can have a significant impact on depression. It also affects the effectiveness of treatments for depression. The current paper presents evidence from two studies in an adolescent and an adult sample indicating that self-compassion acts as a buffer to the impact of maladaptive perfectionism on depression. Individuals with high levels of maladaptive perfectionism are less likely to experience depressive symptoms in the context of high self-compassion. These findings suggest that treatments that help patients cultivate self-compassion might lead to improvements in treatment outcomes for depression, particularly among perfectionistic individuals and further research into these interventions is warranted.

## Supporting information

S1 File Output record of the primary moderation analyses controlling for covariates. (DOCX)

S2 File Study 1 Adolescent de-identified data set. (SAV)

S3 File Study 2 Adult de-identified data set. (SAV)
