# Supplementary material for: Self-compassion moderates the perfectionism and depression link in both adolescence and adulthood
Source: PLoS One. 2018 Feb 21;13(2):e0192022. doi: 10.1371/journal.pone.0192022 (PMC5821438; doi:10.1371/journal.pone.0192022)
Supplement: S1 File — (DOCX) [file pone.0192022.s001.docx]

**Supplementary Information**

**Table 1.**

Hierarchical regression analysis predicting depression (DASS) and probing the interaction between self-compassion (SCS) and maladaptive perfectionism (MPS) in adolescence, controlling for covariates age and gender (n = 527).

|  | *B* | SE *B* | *β* | *sr^2^*^¶^ | *p-*value | 95% CI | VIF |
| --- | --- | --- | --- | --- | --- | --- | --- |
| Model 1 |  |  |  |  |  |  |  |
| Age | 1.08 | 0.39 | 0.12 | 0.12 | .006 | .31, 1.84 | 1.00 |
| Gender | -2.40 | 0.66 | -0.16 | -0.16 | <.0001 | -3.70, -1.10 | 1.00 |
| Model 2 |  |  |  |  |  |  |  |
| Age | 0.29 | 0.30 | 0.03 | 0.04 | .334 | -0.30, 0.87 | 1.02 |
| Gender | -1.75 | 0.50 | -0.11 | -0.15 | .001 | -2.74, -0.76 | 1.01 |
| Self-Compassion | -5.30 | 0.37 | -0.54 | -0.53 | <.0001 | -6.02, -4.57 | 1.32 |
| Maladaptive Perfectionism | 0.12 | 0.02 | 0.19 | 0.22 | <.0001 | 0.07, 0.17 | 1.30 |
| Model 3 |  |  |  |  |  |  |  |
| Age | 0.28 | 0.29 | 0.03 | 0.04 | .334 | -0.29, 0.86 | 1.02 |
| Gender | -1.64 | 0.50 | -0.11 | -0.14 | .001 | -2.61, -0.66 | 1.01 |
| Self-Compassion | -5.50 | 0.37 | -0.56 | -0.55 | <.0001 | -6.22, -4.78 | 1.34 |
| Maladaptive Perfectionism | 0.11 | 0.02 | 0.16 | 0.19 | <.0001 | 0.06, 0.15 | 1.33 |
| Self-Compassion * Maladaptive Perfectionism | -0.14 | 0.03 | -0.15 | -0.19 | <.0001 | -0.20, -0.08 | 1.03 |

Notes: ^¶^*sr^2^* denotes squared semi-partial coefficient. *N* = 527 as 14 participants did not report their age and were excluded from the analysis.

Model 1: *R^2^* = 0.04, *adjusted* *R^2^* =0.04, *F*(2,524) = 10.61, *p* < .001.

Model 2: *R^2^* = 0.45, *adjusted* *R^2^* =0.44, *F*(4,522) = 105.33, *p* < .001.

Model 3: *R^2^* = 0.47, *adjusted* *R^2^* =0.46, *F*(5,521) = 91.30, *p* < .001.

**Table 2.**

Hierarchical regression analysis predicting depression (DASS) and probing the interaction between self-compassion (SCS) and maladaptive perfectionism (MPS) in adulthood, controlling for covariates age and gender (n = 515).

|  | *B* | SE *B* | *β* | *sr^2^*^¶^ | *p-*value | 95% CI | VIF |
| --- | --- | --- | --- | --- | --- | --- | --- |
| Model 1 |  |  |  |  |  |  |  |
| Age | 0.10 | 0.06 | 0.08 | 0.08 | .079 | 0.08, -0.01 | 0.21 |
| Gender | -2.14 | 1.14 | -0.08 | -0.08 | .061 | 0.06, -4.38 | 0.10 |
| Model 2 |  |  |  |  |  |  |  |
| Age | .058 | .044 | .044 | 0.04 | .188 | -0.03, 0.14 | 0.08 |
| Gender | .321 | .880 | .012 | 0.01 | .715 | -1.41, 2.05 | -0.09 |
| Self-Compassion | -6.801 | .687 | -.435 | 0.23 | <.001 | -8.15, -5.45 | -0.62 |
| Maladaptive Perfectionism | .214 | .031 | .300 | -0.33 | <.001 | 0.15, 0.27 | 0.58 |
| Model 3 |  |  |  |  |  |  |  |
| Age | .069 | .043 | .053 | 0.05 | .111 | -0.02, 0.15 | 0.08 |
| Gender | .655 | .867 | .025 | 0.02 | .450 | -1.05, 2.36 | -0.09 |
| Self-Compassion | -7.320 | .684 | -.468 | 0.21 | <.001 | -8.67, -5.98 | -0.62 |
| Maladaptive Perfectionism | .195 | .031 | .274 | -0.35 | <.001 | 0.14, 0.26 | 0.58 |
| Self-Compassion * Maladaptive Perfectionism | -.132 | .029 | -.148 | -0.15 | <.001 | -0.19, -0.08 | -0.11 |

^¶^*sr^2^* denotes squared semi-partial coefficient.

Note: Model 1: *R^2^* = 0.01, *adjusted* *R^2^* = 0.01, *F*(2,512) = 3.48, *p* = 0.032

Model 2: *R^2^* = 0.45, *adjusted* *R^2^* = 0.44, *F*(4,510) = 102.91, *p* < .001

Model 3: : *R^2^* = 0.47, *adjusted* *R^2^* = 0.46, *F*(5,509) = 89.54, *p* < .001
